# Construction of doxycyline-dependent mini-HIV-1 variants for the development of a virotherapy against leukemias

**DOI:** 10.1186/1742-4690-3-64

**Published:** 2006-09-27

**Authors:** Rienk E Jeeninga, Barbara Jan, Henk van den Berg, Ben Berkhout

**Affiliations:** 1 Laboratory of Experimental Virology, Department of Medical Microbiology Center for Infection and Immunity Amsterdam (CINIMA), Academic Medical Center of the University of Amsterdam, Amsterdam, The Netherlands; 2 Department of Paediatric Oncology, Emma Children Hospital, Academic Medical Center of the University of Amsterdam, Amsterdam, The Netherlands

## Abstract

T-cell acute lymphoblastic leukemia (T-ALL) is a high-risk type of blood-cell cancer. We describe the improvement of a candidate therapeutic virus for virotherapy of leukemic cells. Virotherapy is based on the exclusive replication of a virus in leukemic cells, leading to the selective removal of these malignant cells. To improve the safety of such a virus, we constructed an HIV-1 variant that replicates exclusively in the presence of the nontoxic effector doxycycline (dox). This was achieved by replacement of the viral TAR-Tat system for transcriptional activation by the Escherichia coli-derived Tet system for inducible gene expression. This HIV-rtTA virus replicates in a strictly dox-dependent manner. In this virus, additional deletions and/or inactivating mutations were introduced in the genes for accessory proteins. These proteins are essential for virus replication in untransformed cells, but dispensable in leukemic T cells. These minimized HIV-rtTA variants contain up to 7 deletions/inactivating mutations (TAR, Tat, vif, vpR, vpU, nef and U3) and replicate efficiently in the leukemic SupT1 T cell line, but do not replicate in normal peripheral blood mononuclear cells. These virus variants are also able to efficiently remove leukemic cells from a mixed culture with untransformed cells. The therapeutic viruses use CD4 and CXCR4 for cell entry and could potentially be used against CXCR4 expressing malignancies such as T-lymphoblastic leukemia/lymphoma, NK leukemia and some myeloid leukemias.

## Background

Virotherapy has been proposed as a novel therapeutic means against certain cancers and is currently being evaluated in clinical trials [1-3]. This novel strategy is based on the selective replication of viruses in specific target cells to efficiently remove these cells from the patient. Initial successes have been reported in the treatment of head and neck cancers using an engineered adenovirus [4-7], but doubts remain about the absolute restriction of virus replication in cancer cells [8]. In an ideal setting, the therapeutic virus should replicate exclusively in malignant cells. A large number of target cells will enable a fast spreading viral infection at the start of therapy. Consequently, the number of target cells will rapidly decline and result in a concurrent reduction of the virus population. It may be necessary to modify therapeutic viruses to increase their replication specificity and/or to modulate their cytopathogenicity. For instance, cytotoxic genes may be incorporated into the viral genome or virus spread may be improved by inclusion of genes encoding fusogenic proteins [9]. Experiments have thus far focused on virotherapy of solid tumors. Therapeutic viruses have been described based on adenovirus [10,11], herpes simplex virus [12], Newcastle disease virus, poliovirus, vesicular stomatitis virus, measles virus and reovirus [1-3]. No therapeutic viruses have been described that replicate in lymphoid-leukemic cells.

We explored the possibility to use HIV-1 derived viruses, which specifically target T-lymphocytes, as therapeutic virus for leukemia and recently reported the proof of principle with a minimal HIV-1 variant [13]. Our approach was based on the observation that several accessory proteins are not needed for HIV-1 replication in transformed T-cell lines, yet are important for virus replication in primary cells. A minimized derivative of HIV-1 with five gene deletions (vif, vpR, vpU, nef and U3) was demonstrated to replicate in several leukemic T cell lines, but not in normal peripheral blood mononuclear cells (PBMC).

Obvious safety concerns remain for the development of therapeutic viruses based on the human pathogen HIV-1. One of the major concerns is the high mutation and recombination rate of HIV-1 that allows the generation of escape variants over time. For instance, virus evolution frequently leads to the appearance of drug-resistant mutants in patients on antiviral therapy. It could be argued that repair of gene deletions would be impossible, but one cannot exclude alternative viral strategies to improve its fitness or replication capacity. Such an indirect escape strategy has been reported for a HIV-1 vaccine candidate with three gene deletions [14]. Gradual improvement of viral fitness has also been reported for persons infected with a nef-deleted virus variant, coinciding with AIDS disease progression in some of these patients [15]. We therefore designed a method to gain full control over viral replication. For this, we combined the minimal HIV-1 strategy with that of the HIV-rtTA virus [16], a vaccine candidate that was engineered to replicate exclusively in the presence of the nontoxic effector dox. The latter was achieved by replacement of the viral TAR-Tat system for transcriptional activation by the *Escherichia coli*-derived Tet system for inducible gene expression [17]. HIV-rtTA lacks several protein coding genes and non-coding structural elements and replicates in a strictly dox-dependent manner, and has been proposed as a safe form of an attenuated vaccine strain because its replication can be turned on and off at will.

We designed two molecular clones based on HIV-rtTA. rtTAΔ6^A ^carries four deletions (vif, vpR, nef and U3) and two genome regions with inactivating mutations (TAR, vpU). rtTAΔ6^B ^has five deletions (vif, vpR, vpU, nef and U3) and inactivating mutations in TAR. The efficacy of these therapeutic viruses was tested by replication studies in the leukemic T-cell line SupT1 and PBMC. Both viruses replicate efficiently and in a dox-dependent manner in SupT1 cells, resulting in rapid cell killing. In contrast, these viruses are unable to replicate in PBMC. Furthermore, the rtTAΔ6^A ^and rtTAΔ6^B ^viruses were able to selectively infect and remove the SupT1 cells from a mixed culture with PBMC.

## Results

### Design of dox-inducible mini-HIV variants

**Figure 1:**
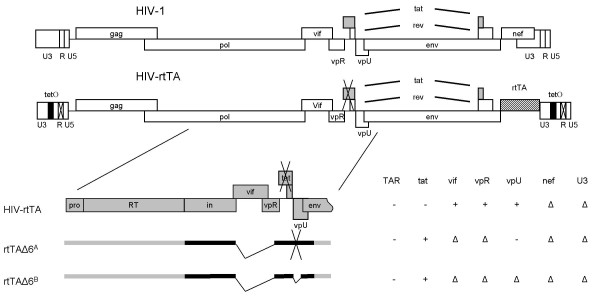
**Overview of the minimal HIV-rtTA molecular clones**. Schematic overview of the different molecular clones used in this study. The position of the various deletions and a summary of the inactivated (-) or deleted (△) viral genes/motifs is provided. See the Materials and Methods section for details on the construction. See also Fig. 7 for further details.

We recently reported the development of a mini-HIV-1 variant for virotherapy of T-ALL [13]. This minimized HIV-1 derivative carries five deletions (vif, vpR, vpU, nef and U3). The deleted genes/motifs contribute to virus replication in untransformed cells, but are dispensable for replication in leukemic T-cells. To obtain control over virus replication, we now combined the mini-HIV approach with the dox-dependent HIV-rtTA concept [16,18]. The nef gene in HIV-rtTA is replaced by the gene encoding the rtTA protein (Fig. 1). In the presence of dox, the transcriptional activator rtTA protein binds to tetO binding sites that were introduced in the U3 domain of the LTR promoter (Fig. 1, black box). Tat-mediated transcriptional activation is abrogated by an inactivating mutation in the tat gene (Tyr26Ala)[19,20] and multiple inactivating mutations in the TAR hairpin (indicated by crosses in Fig. 1) [16]. HIV-rtTA also carries a deletion of a large upstream part of the U3 domain [21] and thus represents a Δ4 HIV-rtTA genome (Fig. 1, ΔTAR, tat, nef, U3). HIV-rtTA was further minimized by deletion of genes encoding the accessory proteins Vif and VpR. Additionally, the vpU gene was inactivated in rtTAΔ6^A ^(Fig. 1) by mutation of the startcodon (AUG to AUA) and in rtTAΔ6^B ^by gene deletion. Due to the vpU-cloning procedure, the wild type tat gene was restored. These minimized rtTAΔ6^A ^and rtTAΔ6^B ^variants express the basic set of HIV-1 proteins (gag, pol, env), the essential Rev and Tat proteins, but lack the accessory proteins Vif, VpR, VpU and Nef. The RNA genome of rtTAΔ6^B ^is 8,872 nt compared to 9,229 nt for full length HIV-1 LAI and 9,607 nt for the parental HIV-rtTA virus.

### Replication characteristics of the mini-rtTA viruses

**Figure 2:**
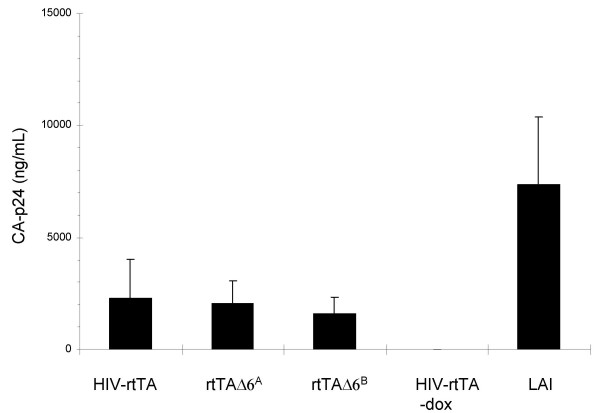
**Virus production of HIV-rtTA constructs**. The non-susceptible C33A cell line was transfected with five microgram of the indicated plasmids. The culture supernatant was harvested after three days and used in a CA-p24 Elisa to determine virus production. All rtTA samples were cultured with 1000 ng/mL dox unless indicated otherwise. The figure is representative for three independent transfections.

Viral gene expression and production of virus particles was tested by transfection of the mini-rtTA plasmids in C33A cells. These cells lack the CD4 receptor and are thus not susceptible for multiple rounds of HIV-1 replication. We measured no difference in virus production of the mini-rtTA viruses compared with the original HIV-rtTA construct (Fig. 2). All constructs are fully dependent on dox for gene expression. These results demonstrate that none of the deleted/mutated genes/motifs play an important role in viral gene expression (transcription, splicing, and translation) and the assembly of new virions. Virus production of all dox-dependent rtTA viruses is somewhat lower than that of the wild type LAI virus, consistent with our previous studies [16,22].

**Figure 3:**
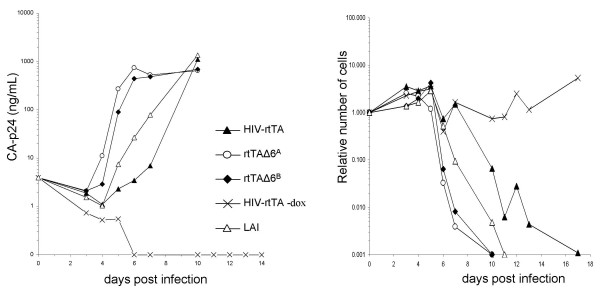
**Replication and cell killing capacity of dox-inducible viruses in SupT1 cells**. (**Left**) Virus replication of LAI (△), HIV-rtTA (▲), HIV-rtTAΔ6^A ^(○), HIV-rtTAΔ6^B ^(◆) and HIV-rtTA without dox (×) was determined by measuring of the supernatant CA-p24 concentration after infection with virus (20 ng CA-p24) in a 5 mL SupT1 culture. (**Right**) The number of cells in each culture was determined by a 30 sec time limit FACS analysis. The cell killing capacity of the viruses was determined as the ratio of SupT1 cells present in the infected culture versus the uninfected control culture.

The virus stocks produced in C33A cells were used to infect the HIV-susceptible leukemic T-cell line SupT1. Virus replication was followed by sampling of the culture supernatant and measurement of the CA-p24 concentration (Fig. 3, left panel). Surprisingly, replication of the minimized rtTAΔ6^A ^and rtTAΔ6^B ^variants is significantly faster than that of the parental HIV-rtTA virus and even faster than the wild type LAI virus. Similar results were obtained in multiple replication assays that were initiated either by virus infection or by transfection of the molecular clones (results not shown). Direct virus competition assays confirmed this ranking order, with rtTAΔ6^A ^being slightly more fit than rtTAΔ6^B^, and both much more fit than HIV-rtTA (results not shown). This surprise finding will be dealt with in detail later in this paper. As expected, virus replication is fully dependent on dox addition.

The T-cell cultures were also analyzed for the cell killing capacity of these viruses. A time-limited FACS analysis was used to determine the relative number of live cells in the infected cultures and a mock-infected SupT1 culture as the control. The cell killing capacity was determined by dividing the number of cells in the infected culture by the number of cells in the control culture (Fig. 3, right panel). The LAI virus and the different HIV-rtTA variants are able to kill all SupT1 cells. The cell killing kinetics correlate nicely with the replication capacity of the respective viruses. There was no decrease in the number of live cells when the HIV-rtTA virus was tested without dox, confirming that the increase in cell death is the result of active virus replication.

We tried to set up experiments with patient derived primary leukemic T-cells but the high death rate of these cells in in vitro culture experiments (without any virus) prevented any significant conclusions to be reached about virus-induced cell killing (results not shown).

### Switching virus replication on and off at will

**Figure 4:**
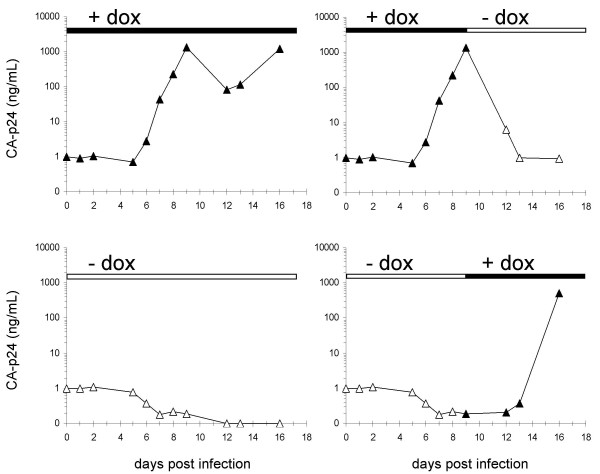
**Dox regulated replication of the mini-rtTA virus rtTAΔ6^A^**. SupT1 cells were infected with rtTAΔ6^A ^virus (1 ng CA-p24). The culture was split and the cells were cultured with dox (upper panels) or without (lower panels). Virus replication was monitored by CA-p24 Elisa on the culture supernatant. At day 9 post infection, both cultures were washed and each culture split into one culture with dox (left panels) and one without (right panels). Filled triangles indicate cultures without dox and open triangles indicate cultures with 1000 ng/mL dox.

Dox-regulation should allow strict control over replication of the therapeutic viruses. To demonstrate the regulatory possibilities of this system, we followed several rtTAΔ6^A ^cultures with different dox regimens, ranging from no to continuous dox treatment. We also tested delayed dox addition and dox-withdrawal near the peak of infection. Virus infections were started with dox (Fig. 4, upper left panel) or without dox (Fig. 4, lower left panel) and virus production was followed by measurement of the CA-p24 concentration in the supernatant. After nine days, the cultures were split and either continued with the same treatment (Fig. 4, left panels) or switched from dox to no dox (Fig. 4, upper right panel) or vice versa (Fig. 4, lower right panel). The results show that virus replication is completely controllable by dox. In the cultures with dox a productive infection is started that can be turned off by withdrawal of dox. In the cultures that were started without dox, a single round of infection takes place that leads to the establishment of an integrated but silent provirus, which can subsequently be activated by the addition of dox.

### Replication characteristics of the HIV-rtTA viruses in PBMC

**Figure 5:**
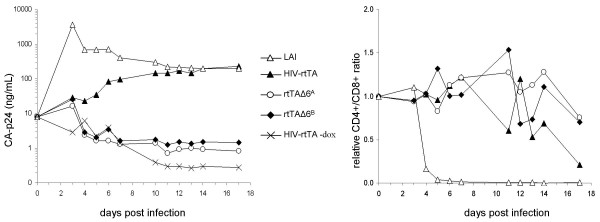
**Replication and cell killing capacity of dox inducible viruses in PBMC**. (**Left**) Virus replication of LAI (△), HIV-rtTA (▲), rtTAΔ6^A ^(○), rtTAΔ6^B ^(◆) and rtTA without dox (×) was determined by monitoring the supernatant CA-p24 concentration after virus infection (40 ng CA-p24) in a 5 mL PBMC culture. (**Right**) The CD4+ and CD8+ cell populations in the infected cultures and an uninfected control culture were quantified by a 30 sec time limit FACS analysis and the CD4/CD8 ratio was calculated. The figure shows the CD4/CD8 ratio in the infections normalized for the control uninfected PBMC culture.

The different HIV-rtTA viruses were further analyzed by testing their replication capacity on PBMC (Fig. 5, left panel). Killing of the CD4+ target cells was plotted as the CD4+/CD8+ ratio relative to that of the control culture without dox (Fig. 5, right panel). The wild type HIV-1 LAI isolate replicates efficiently, resulting in a high peak of CA-p24 production and complete removal of the CD4+ cells from the PBMC culture within 5 days. Due to the removal of target cells, the CA-p24 concentration reaches a maximum at 3 days post infection and subsequently levels off. The parental HIV-rtTA virus replicates slowly, but eventually reaches CA-p24 values similar to that of the wt virus. In this culture, a gradual reduction in CD4+ cells is scored, but HIV-rtTA replication is completely dependent on dox addition. No production of CA-p24 was measured in the PBMC cultures infected with the minimized rtTAΔ6^A ^and rtTAΔ6^B ^variants, and no significant reduction in the CD4+/CD8+ ratio was observed. Thus, these viruses are unable to cause a spreading infection in PBMC. Extending the time for replication by feeding these cultures with fresh PBMC did also not result in a spreading infection.

It cannot be excluded that the rtTAΔ6^A ^and rtTAΔ6^B ^viruses replicate at an extremely low level, and thus stay below the CA-p24 detection limit. To test for this, we used a very sensitive SupT1-based rescue assay to screen for viable virus in the PBMC cultures. PBMC were harvested at day 13, washed and subsequently co-cultured with SupT1 cells. Virus replication is readily observed in the control co-cultures derived from the LAI and HIV-rtTA infections. No virus could be detected in the cultures derived from the rtTAΔ6^A ^or rtTAΔ6^B ^infections, even with 1000-fold more input sample compared to the LAI or HIV-rtTA samples.

### Selective removal of leukemic T-cells from a mixed culture

**Figure 6:**
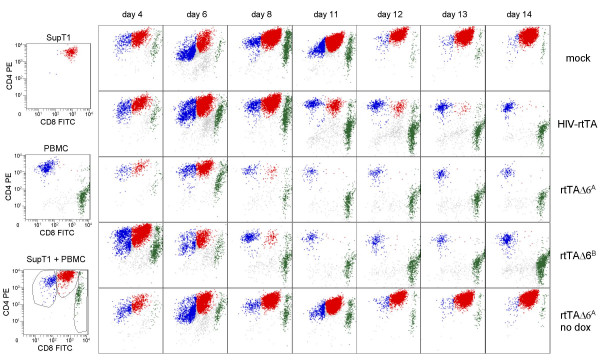
**Selective SupT1 killing in SupT1/PBMC co-cultures by mini-rtTA viruses**. Infections were started with virus corresponding to 40 ng CA-p24 or mock infected. The FACS dot plot of the initial PBMC + SupT1 cell mixture is in the lower left corner, and the separate cultures are shown above. The gates for CD4+ PBMC (blue), SupT1 (red) and CD8+ PBMC (green) are indicated. The composition of the PBMC + SupT1 culture was followed over time upon infection with the indicated viruses.

In a virotherapy setting, the blood of a patient will contain a mixture of leukemic and untransformed cells. The viral therapeutic agent should selectively replicate and kill the leukemic target cells without affecting the untransformed cells. To mimic this situation in our *in vitro *culture system, we started co-cultures of the SupT1 cell line and PBMC. These cells can easily be distinguished by FACS analysis using the CD4 and CD8 surface markers. SupT1 cells are double positive T-cells (CD4^+^CD8^+^), whereas PBMC contain a mixture of single positive CD4^+^CD8^- ^and CD4^-^CD8^+ ^cells (Fig. 6, left). A PBMC-SupT1 culture was split in five samples. These cultures were infected with an equal amount of HIV-rtTA, rtTAΔ6^A ^or rtTAΔ6^B ^virus. The two remaining cultures were used for a mock infection and a control rtTAΔ6^A ^infection without dox. The cell composition was followed over time by FACS analysis, showing the more rapid proliferation of leukemic SupT1 cells versus PBMC in the uninfected control (mock, upper panels). The same result was obtained for the rtTAΔ6^A ^control without dox (rtTAΔ6^A^-dox, lower panels). In contrast, the SupT1 cells are selectively depleted in 8 days from the cultures containing rtTAΔ6^A ^or rtTAΔ6^B ^virus with dox. In agreement with the slower replication kinetics of HIV-rtTA in SupT1 cells (Fig. 3), SupT1-depletion is delayed for this virus. These results indicate that it is possible to selectively remove leukemic T-cells from a mixture with untransformed cells by the use of a dox-controlled mini-HIV-1 variant.

### Effects of different Tat proteins on the replication of the dox-inducible mini-rtTA viruses

We constructed two dox-regulated viruses that specifically target leukemic T cells. A surprising finding was that these viruses, with many deletions (Δ6), replicated much better in SupT1 cells than the parental construct HIV-rtTA (Fig. 3). In the construction of rtTAΔ6^A ^and rtTAΔ6^B^, the wild-type tat open reading frame is restored when compared to the rtTA virus that carries the Y26A inactivating Tat mutation. Although Tat-mediated transcriptional activation is not needed for replication of the dox-controlled virus, it is possible that Tat restoration enhances virus replication by other means, which may explain the enhanced replication of rtTAΔ6 variants.

To test this hypothesis, the Y26A mutation was reintroduced in the rtTAΔ6^A ^and rtTAΔ6^B ^background, yielding the rtTAΔ7^A ^and rtTAΔ7^B ^viruses, respectively. For comparison, the mutant tat gene (Y26A) in the HIV-rtTA virus was also replaced by the wild-type tat gene from the LAI isolate, yielding rtTAΔ3 (LAI), or the tat gene from the NL4-3 isolate, yielding rtTAΔ3 (NL4-3). This set of viruses was used to infect SupT1 cells to test their replication capacity (Fig. 8). Comparison of the replication capacity of rtTAΔ6^A ^versus rtTAΔ7^A^, rtTAΔ6^B ^versus rtTAΔ7^B ^(Fig. 8, left) and rtTAΔ3 (LAI) versus HIV-rtTA (Fig. 8, right) demonstrate that the Y26A Tat mutation causes a small decrease in replication. Thus, a wild-type tat gene improves replication. The introduction of the NL4-3 tat gene in HIV-rtTA, however, improved replication much more than introduction of the tat gene of the LAI isolate (Fig. 8, left panel, compare rtTAΔ3 (NL4-3) with HIV-rtTA and rtTAΔ3 (LAI)). In fact, the rtTAΔ3 (NL4-3) variant replicated consistently better that the wild type LAI virus. Similar results were obtained in repeated infections and in replication studies that were initiated by DNA transfection (results not shown).

**Figure 7:**
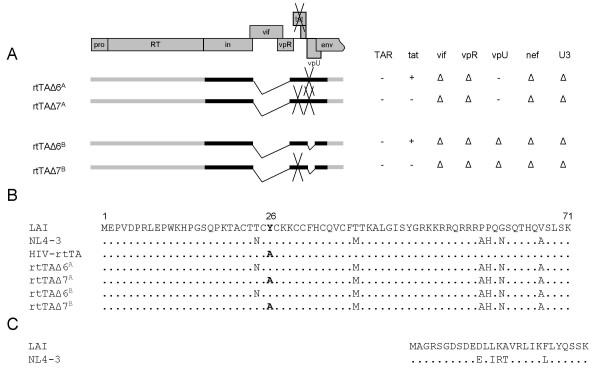
**Overview of the different tat constructs**. (**A**) The position of the various deletions and mutations, and a summery of the inactivated (-) or deleted (△) viral genes is shown. See the Materials and Methods section for construction details. (**B**) Sequence alignment of the different tat genes. The position of the Y26A mutation is indicated in bold. (**C**) Sequence alignment of the corresponding part of the rev gene. The rev startcodon overlaps the tat codon for Y47.

**Figure 8:**
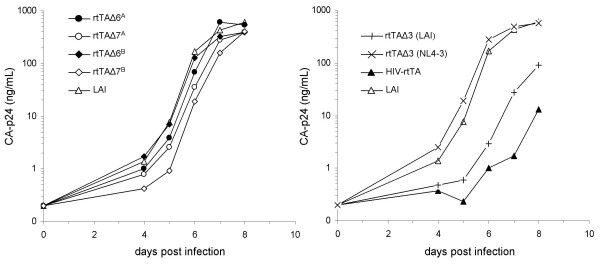
**Effects of different Tat proteins on the replication of the dox inducible rtTA viruses**. Virus replication was followed by measuring of the supernatant CA-p24 concentration after virus infection (20 ng CA-p24) in a 5 mL culture. (**Left**) Replication of the rtTAΔ6^A ^(●), rtTAΔ7^A ^(○), rtTAΔ6^B ^(◆), rtTAΔ7^B ^(◇) and LAI (△) viruses in Sup T1 cells. (**Right**) Replication of the rtTAΔ3 (LAI, +), rtTAΔ3 (NL4-3, X), HIV-rtTA (▲) and LAI (△) viruses in Sup T1 cells.

## Discussion

We describe the development of therapeutic viruses based on HIV-1 for virotherapy against T-ALL. We combined mini-HIV-1 variants [13] that lack several accessory proteins with the dox-controllable HIV-rtTA virus approach [16]. One molecular clone, rtTAΔ6^A^, has four deletions (vif, vpR, nef and U3) and two motifs with inactivating mutations (TAR and vpU). The molecular clone rtTAΔ6^B ^is similar, but the vpU gene is deleted instead of having the inactivating mutation. These mini-rtTA viruses replicate efficiently in leukemic T-cell lines and virus replication results in cell death. These viruses do not replicate in PBMC, even in co-cultures with susceptible SupT1 cells that continuously produce new infectious virus particles (Fig. 6). The results are summarized in Table 1. Most importantly, virus replication is strictly dox-dependent.

**Table 1:** Virus replication and cell killing capacity

	SupT1		PBMC	
virus	replication	cell killing	replication	CD4+ killing

LAI	++	++	++	++
HIV-rtTA	+	++	+	±
rtTAΔ6A	++	++	-	-
rtTAΔ6B	++	++	-	-

The viral Vif protein counters the potent antiviral activity of APOBEC3G in some cells including PBMC [reviewed in [23]], and the absence of Vif may therefore be the main contributor to the replication defect in primary cells. Nevertheless, the other accessory proteins (vpR, vpU and Nef) also have important roles in vivo [24-26] and in vitro [13,27-29]. The presence of multiple gene deletions will not only increase safety of the therapeutic virus, but may also provide synergistic effects. For instance, it was recently demonstrated that the combined elimination of the vif and vpR genes, unlike the individual mutants, renders the virus incapable of causing cell death and G2 cell cycle arrest [30].

A surprising finding is that removal of the genes encoding the accessory proteins Vif, VpR and VpU appeared to have a positive effect in the context of the dox-controlled HIV-rtTA virus, whereas the same deletions have a negative impact when introduced into the wild-type HIV-1 isolate [13]. This observation enabled us to make HIV-1 variants that replicate extremely fast in leukemic cells, yet are fully replication-impaired in primary cells. This result, combined with the strict dox-regulation, suggests to us that a safe therapeutic use of these virus variants is feasible. In a therapeutic setting, the minimized virus can be used to target the leukemic cells in the presence of dox. This will result in a self-limiting viral infection since the target cells are killed by the virus. Withdrawal of dox provides an additional safety feature to block ongoing replication after the leukemic cells are removed. It may be possible to add therapeutic short interfering RNAs (siRNAs) to this viral vector system [31]. We plan to set up a T-ALL model in severe combined immunodeficiency (SCID) mice to test the capacity of these therapeutic viruses to selectively remove leukemic cells *in vivo*.

HIV-rtTA was originally designed as a novel attenuated virus vaccine candidate. To minimize the possibility of reversion to normal TAR-Tat regulated transactivation, inactivating mutations were made in both the TAR hairpin and the Tat protein (Y26A). In our minimized Δ6 deletion variants, a wild type NL4-3 tat gene was introduced due to the cloning procedure. Restoration of a wild type Tat function could explain the observed fast replication kinetics of these viruses. However, reintroduction of the Y26A mutation in these viruses (rtTAΔ7^A ^and rtTAΔ7^B^) caused only a small decrease in replication capacity, which is consistent with previous results [16]. The TAR hairpin in these constructs is inactivated by multiple point mutations, which are sufficient as individual point mutation to block Tat-mediated transcription [16,32-34] and virus replication [35]. Restoration of the normal Tat-TAR transcription axis is therefore an unlikely scenario in the dox-dependent virus. Thus, the absence of the Y26A mutation does not provide an explanation for the improved replication, but the results demonstrate that the Y26A mutation, apart from abolishing Tat-TAR mediated transcription, has an additional (small) negative effect on the replication of HIV-rtTA.

Another possible explanation for the improved replication of the mini-HIV-rtTAs is provided by inspection of the sizes of these viral genomes. The RNA genome of the wild-type HIV-1 LAI isolate is 9,229 nt, but the HIV-rtTA genome is extended to 9,607 nt due to the insertion of the rtTA gene and tetO DNA binding sites. The latter genome size may be sub-optimal for replication, *e.g*. due to restricted RNA packaging in virion particles, and removal of the vif-vpR-vpU genes may thus be beneficial in this context. Deletion of these genes reduces the RNA genome to 8989 for rtTAΔ6^A ^and to 8872 for rtTAΔ6^B^. One would nevertheless expect a reduction of viral fitness due to removal of three accessory genes, unless these viral-protein functions do not add significantly to virus replication in T cell lines. In fact, we consistantly measured that the rtTAΔ6 variants replicate significantly faster than the wild-type virus in T-cell lines, perhaps indicating that some of the accessory HIV-1 genes have a negative impaction on virus replication in these leukemic cells. Consistent with this idea is the frequent selection of inactivation mutations in these open reading frames upon prolonged culturing in T cell lines. Alternatively, these viral functions may have lost significance in the context HIV-rtTA, in which Tat-TAR mediated transcription is taken over by the rtTA-tetO elements. For instance, VpR has been reported to have a transcriptional component [36], and this transcriptional contribution may be less important in the HIV-rtTA context.

Another explanation comes from the comparison of the control viruses rtTAΔ3 (NL4-3) and rtTAΔ3 (LAI) that have the same gene deletions, yet a different tat gene. The introduction of the NL4-3 tat gene improved virus replication significantly more than insertion of the LAI tat gene. In fact, the replication of rtTAΔ3 (NL4-3) is similar to that of rtTAΔ6^A ^and rtTAΔ6^B^. Thus, the presence of a fragment encoding the NL4-3 tat gene is the decisive determinant for the improved replication of rtTAΔ6^A^, rtTAΔ6^B ^and rtTAΔ3 (NL4-3). As discussed above, this is not due to the Y26A mutation, which has a similar small negative effect in both sequence contexts (LAI and NL4-3). Furthermore, this effect appears to be specific for the HIV-rtTA virus since replication of the mini-HIV-1 virus, which has a wild type NL4-3 tat gene, is impaired [13].

The differences between the fast replicating virus rtTAΔ3 (NL4-3) and the slow replicating rtTAΔ3 (LAI) are located exclusively in the 350 nt tat fragment. This fragment encodes the first exon of the tat gene, the overlapping first exon of the rev gene and part of the open reading frames for vpU and Env. The sequence differences result in six amino acid substitutions in Tat (Fig. 7B, N24T in the Cysteine-rich domain, M39T in the core domain and A58P, H59P, N61G and A67V in the C-terminal domain). In addition, these sequence differences also change the rev gene (Fig. 7C, E11D, I13L, R14K T15A and L21F). Furthermore, there are two substitutions in the vpU gene (I5Q and V60I). We can exclude some of these differences to play a role in this phenotype by comparison with the efficient replicating rtTAΔ7^A ^and rtTAΔ7^B ^viruses. These viruses lack the vpU gene and have the LAI-specific Threonine at position 24 in Tat, indicating that these motifs are not responsible for the improved phenotype. Thus, the differences are caused by one or more of the remaining substitutions in the core and/or C-terminal domain of Tat or the overlapping Rev protein. Recently, it was reported that tat genes from different HIV-1 subtypes differentially regulate gene expression [37]. Our results demonstrate that sequence variation in this genome segment can have a profound effect on replication even when derived from the same subtype B.

## Materials and methods

### DNA-constructs

Full-length molecular HIV-1 clones are based on an improved variant of the dox-inducible HIV-1 variant described previously [38]. We first deleted the accessory proteins vif and vpR in this HIV-rtTA virus. Plasmid pDR2483 [39], which contains the 5' genome of the HIV-1 isolate NL4-3 with deletions in the genes encoding the vif and vpR proteins, was used as template in a PCR reaction with primers RJ001 (5' GGG CCT TAT CGA TTC CAT CTA 3') and 6 N (5'CTT CCT GCC ATA GGA GAT GCC TAA G 3'). The resulting PCR fragment was cut with *Cla*I and *Eco*R1 and ligated with a 9644 bp *Bcl*I-*Eco*R1 HIV-rtTA vector fragment and a 1816 bp *Bcl*I-*Cla*I fragment from pLAI-001 [13] to generate the subclone rtTAΔvifΔvpR. We noticed a vpU startcodon inactivation (AUG to AUA) in one of the evolution cultures [13]. Proviral DNA was PCR amplified from total cellular DNA of this culture with the primers Pol5'FM (5'TGG AAA GGA CCA GCA AAG CTC CTC TGG AAA GGT 3') and WS3 (5'TAG AAT TCA AAC TAG GGT ATT TGA CTA AT). The same PCR was performed on DNA from a vpU-deletion construct [pDR2484, [39]]. The PCR fragments were cut with *Eco*RI and *Nde*I and ligated with a 2086 bp wild type (wt)t rtTA *Bam*HI-*Nde*I fragment and the vector rtTAΔvifΔvpR cut with *Eco*RI and *Bam*HI. The resulting molecular clones (Fig. 1) were named rtTAΔ6^A ^(vpU startcodon inactivation) and rtTAΔ6^B ^(vpU deletion).

As part of the vpU inactivation strategy, the Y26A inactivating mutation in the tat gene of HIV-rtTA is replaced by the wt tat gene of the NL4-3 isolate (first exon). The Y26A mutation was cloned back into the rtTAΔ6^A ^and rtTAΔ6^B ^molecular clones as follows. A PCR was done with HIV-rtTA as template and primers Pol5'FM and RJ036 (5'CTT TTG TCA TGA AAC AAA CTT GGC A 3'). The latter primer introduces a *Bsp*HI site that is also present in the wt NL4-3 sequence. The PCR product was digested with *Eco*RI and *Bsp*HI and used in a triple ligation with the 9028 bp *Eco*RI-*Bam*HI vector and either the 2545 bp *Bsp*HI-*Bam*HI fragment of rtTAΔ6^A ^or the 2428 bp *Bsp*HI-*Bam*HI fragment of rtTAΔ6^B^. For comparison, we also introduced the LAI and NL4-3 tat gene into the HIV-rtTA background. For NL4-3, this was done in a triple ligation with the rtTA vector cut with *Sph*I and *Asp718 *I, the 4378 bp *Sal*I-*SpH*I rtTA fragment and the 558 bp *Sal*I-*Asp718 *I fragment of pDR2480 [39]. For LAI this was done by ligation of the 9646 bp *Nco*I-*Bam*HI digested HIV-rtTA vector with the 2811 bp *Nco*I-*Bam*HI fragment of LAI.

All constructs were verified by restriction enzyme digestion and BigDye terminator sequencing (Applied Biosystems, Foster City, CA) with appropriate primers on an automatic sequencer (Applied Biosystems DNA sequencer 377). Plasmid DNA isolation was done with the Qiagen Plasmid isolation kit according to the manufacturers' protocol (Qiagen, Chatsworth, CA).

### CA-p24 levels

Culture supernatant was heat inactivated at 56°C for 30 min in the presence of 0.05% Empigen-BB (Calbiochem, La Jolla, USA). CA-p24 concentration was determined by a twin-site ELISA with D7320 (Biochrom, Berlin, Germany) as the capture antibody and alkaline phosphatase-conjugated anti-p24 monoclonal antibody (EH12-AP) as the detection antibody. Detection was done with the lumiphos plus system (Lumigen, Michigan, USA) in a LUMIstar Galaxy (BMG labtechnologies, Offenburg, Germany) luminescence reader. Recombinant CA-p24 expressed in a baculovirus system was used as the reference standard.

### Cells and viruses

C33A cervix carcinoma cells [ATCC HTB31, [40]] were grown as a monolayer in Dulbecco's minimal essential medium supplemented with 10% (v/v) fetal calf serum (FCS), 100 U/mL penicillin, 100 μg/mL streptomycin, 20 mM glucose and minimal essential medium nonessential amino acids at 37°C and 5% CO_2_. The cells were transfected by the calcium phosphate method as described previously [41].

The human T lymphocyte cell line SupT1 [ATCC CRL-1942, [42]] was cultured in RPMI 1640 (Gibco BRL, Gaithersburg, MD) supplemented with 10% (v/v) FCS, 100 U/mL penicillin, and 100 μg/mL streptomycin at 37°C and in 5% CO_2_. Transfections were carried out by electroporation as described [43] using a BioRad Gene Pulser II (BioRad, Hercules, CA). Infection with C33A produced virus stocks was performed with the indicated amount of virus.

PBMC were isolated from different healthy donors, each batch consists of a mixture of four different donors. PBMC were grown as the SupT1 cells, but with the addition of 100 U/mL human IL2 after an initial PHA (5 μg/mL) stimulation for 2 days. Infections were performed with C33A produced virus stocks with the indicated amount of virus.

### Virus competition assay

Virus competition experiments were done as described previously [44]. Competitions were initiated with C33A produced virus stocks. Each competition was done with virus corresponding to 6 ng virus with starting ratios of 5 to 1, 1 to 1 and 1 to 5. The competition was repeated with independent virus stocks.

### Virus rescue assay

Low-level replication in PBMC was analyzed with a virus rescue assay. At day 13 post infection of PBMC, the cells from 1 mL culture were collected (4 min 4000 RPM, eppendorf centrifuge), washed once with 1 mL PBS to remove any input virus and resuspended in medium. A dilution series (1, 10×, 100×, 1000×, 10.000×) was made and each sample mixed with one million SupT1 cells. The cultures were maintained for four weeks, regularly split and inspected for virus replication by CA-p24 Elisa on the culture supernatant and visual inspection for syncytia formation.

### Fluorescence-activated cell sorting analysis

Flow cytometry was performed with RPE-conjugated mouse monoclonal anti-human CD4 (clone MT310, Dako, Glostrup, Denmark) and FITC-conjugated mouse monoclonal anti-human CD8 (clone DK25, Dako). Cells from a 1 mL culture sample were collected (4 min 4000 RPM, eppendorf centrifuge), incubated with a mixture of both monoclonal antibodies in fluorescence-activated cell sorting (FACS) buffer (PBS with 2% FCS) for 30 min at room temperature and washed with 800 μL FACS buffer. The cells were subsequently collected (4 min 4000 RPM, eppendorf centrifuge) and resuspended in 20 μL of 4% paraformaldehyde. After 5-minute incubation at room temperature, 750 μL FACS buffer was added and the suspension analyzed on a FACScalibur flow cytometer with CellQuest Pro software (BD biosciences, San Jose, CA). The machine was set for a 30-sec collection time. Cell populations were defined based on forward/sideward scattering and isotype controls were used to set markers. For mixed SupT1 plus PBMC cultures, the gates for PBMC (CD4^+^CD8^- ^and CD4^-^CD8^+^) and SupT1 (CD4^+^, CD8^+^) were set using a separate control culture.

### Mixed culture SupT1/PBMC infection

Freshly isolated PBMC were stimulated for 2 days with PHA (5 μg/mL), washed twice with medium and mixed with SupT1 cells. The cell mixture was analyzed by FACS staining for CD4 and CD8 as described above. The culture was divided into equal 10 mL samples, containing approximately 1 million PBMC and 2 million SupT1 cells, which were infected with different virus variants (input 40 ng CA-p24). Daily samples were taken for CA-p24 Elisa and anti-CD4/CD8 FACS analysis.

## Competing interests

The author(s) declare that they have no competing interests.

## References

[B1] Ring CJ (2002). Cytolytic viruses as potential anti-cancer agents. J Gen Virol.

[B2] Parato KA, Senger D, Forsyth PA, Bell JC (2005). Recent progress in the battle between oncolytic viruses and tumours. Nat Rev Cancer.

[B3] Lin E, Nemunaitis J (2004). Oncolytic viral therapies. Cancer Gene Ther.

[B4] Bischoff JR, Kirn DH, Williams A, Heise C, Horn S, Muna M, Ng L, Nye JA, Sampson-Johannes A, Fattaey A, McCormick F (1996). An adenovirus mutant that replicates selectively in p53-deficient human tumor cells. Science.

[B5] Nemunaitis J, Cunningham C, Tong AW, Post L, Netto G, Paulson AS, Rich D, Blackburn A, Sands B, Gibson B, Randlev B, Freeman S (2003). Pilot trial of intravenous infusion of a replication-selective adenovirus (ONYX-015) in combination with chemotherapy or IL-2 treatment in refractory cancer patients. Cancer Gene Ther.

[B6] Nemunaitis J, Ganly I, Khuri F, Arseneau J, Kuhn J, McCarty T, Landers S, Maples P, Romel L, Randlev B, Reid T, Kaye S, Kirn D (2000). Selective replication and oncolysis in p53 mutant tumors with ONYX-015, an E1B-55kD gene-deleted adenovirus, in patients with advanced head and neck cancer: a phase II trial. Cancer Res.

[B7] Khuri FR, Nemunaitis J, Ganly I, Arseneau J, Tannock IF, Romel L, Gore M, Ironside J, MacDougall RH, Heise C, Randlev B, Gillenwater AM, Bruso P, Kaye SB, Hong WK, Kirn DH (2000). A controlled trial of intratumoral ONYX-015, a selectively-replicating adenovirus, in combination with cisplatin and 5-fluorouracil in patients with recurrent head and neck cancer. Nat Med.

[B8] Dix BR, Edwards SJ, Braithwaite AW (2001). Does the antitumor adenovirus ONYX-015/dl1520 selectively target cells defective in the p53 pathway?. J Virol.

[B9] Li H, Haviv YS, Derdeyn CA, Lam J, Coolidge C, Hunter E, Curiel DT, Blackwell JL (2001). Human immunodeficiency virus type 1-mediated syncytium formation is compatible with adenovirus replication and facilitates efficient dispersion of viral gene products and de novo-synthesized virus particles. Hum Gene Ther.

[B10] Sarkar D, Su ZZ, Vozhilla N, Park ES, Randolph A, Valerie K, Fisher PB (2005). Targeted virus replication plus immunotherapy eradicates primary and distant pancreatic tumors in nude mice. Cancer Res.

[B11] Sarkar D, Su ZZ, Vozhilla N, Park ES, Gupta P, Fisher PB (2005). Dual cancer-specific targeting strategy cures primary and distant breast carcinomas in nude mice. Proc Natl Acad Sci U S A.

[B12] Gillet L, Dewals B, Farnir F, de Leval L, Vanderplasschen A (2005). Bovine herpesvirus 4 induces apoptosis of human carcinoma cell lines in vitro and in vivo. Cancer Res.

[B13] Jeeninga RE, Van der Linden B, Jan B, Van den Berg H, Berkhout B (2005). Construction of a minimal HIV-1 variant that selectively replicates in leukemic derived T-cell lines: towards a new virotherapy approach. Cancer Res.

[B14] Berkhout B, Verhoef K, van Wamel JLB, Back B (1999). Genetic instability of live-attenuated HIV-1 vaccine strains. J Virol.

[B15] Birch MR, Learmont JC, Dyer WB, Deacon NJ, Zaunders JJ, Saksena N, Cunningham AL, Mills J, Sullivan JS (2001). An examination of signs of disease progression in survivors of the Sydney Blood Bank Cohort (SBBC). J Clin Virol.

[B16] Verhoef K, Marzio G, Hillen W, Bujard H, Berkhout B (2001). Strict control of human immunodeficiency virus type 1 replication by a genetic switch: Tet for Tat. J Virol.

[B17] Berkhout B, Verhoef K, Marzio G, Klaver B, Vink M, Zhou X, Das AT (2002). Conditional virus replication as an approach to a safe live attenuated human immunodeficiency virus vaccine. J Neurovirol.

[B18] Marzio G, Verhoef K, Vink M, Berkhout B (2001). In vitro evolution of a highly replicating, doxycycline-dependent HIV for applications in vaccine studies. Proc Natl Acad Sci USA.

[B19] Verhoef K, Koper M, Berkhout B (1997). Determination of the minimal amount of Tat activity required for human immunodeficiency virus type 1 replication. Virol.

[B20] Verhoef K, Berkhout B (1999). A second-site mutation that restores replication of a Tat-defective human immunodeficiency virus. J Virol.

[B21] Das AT, Verhoef K, Berkhout B (2004). A conditionally replicating virus as a novel approach toward an HIV vaccine. Methods Enzymol.

[B22] Marzio G, Vink M, Verhoef K, de Ronde A, Berkhout B (2002). Efficient human immunodeficiency virus replication requires a fine-tuned level of transcription. J Virol.

[B23] Cullen BR (2006). Role and mechanism of action of the APOBEC3 family of antiretroviral resistance factors. J Virol.

[B24] Rhodes DI, Ashton L, Solomon A, Carr A, Cooper D, Kaldor J, Deacon N (2000). Characterization of three nef-defective human immunodeficiency virus type 1 strains associated with long-term nonprogression. Australian Long-Term Nonprogressor Study Group. J Virol.

[B25] Deacon NJ, Tsykin A, Solomon A, Smith K, Ludford-Menting M, Hooker DJ, McPhee DA, Greenway AL, Ellett A, Chatfield C, Lawson VA, Crowe S, Maerz A, Sonza S, Learmont J, Sullivan JS, Cunningham A, Dwyer D, Dowton D, Mills J (1995). Genomic structure of an attenuated quasi species of HIV-1 from blood transfusion donor and recipients. Science.

[B26] Lum JJ, Cohen OJ, Nie Z, Weaver JG, Gomez TS, Yao XJ, Lynch D, Pilon AA, Hawley N, Kim JE, Chen Z, Montpetit M, Sanchez-Dardon J, Cohen EA, Badley AD (2003). Vpr R77Q is associated with long-term nonprogressive HIV infection and impaired induction of apoptosis. J Clin Invest.

[B27] James CO, Huang MB, Khan M, Garcia-Barrio M, Powell MD, Bond VC (2004). Extracellular Nef protein targets CD4+ T cells for apoptosis by interacting with CXCR4 surface receptors. J Virol.

[B28] de Ronde A, Klaver B, Keulen W, Smit L, Goudsmit J (1992). Natural HIV-1 NEF accelerates virus replication in primary human lymphocytes. Virol.

[B29] Somasundaran M, Sharkey M, Brichacek B, Luzuriaga K, Emerman M, Sullivan JL, Stevenson M (2002). Evidence for a cytopathogenicity determinant in HIV-1 Vpr. Proc Natl Acad Sci U S A.

[B30] Sakai K, Dimas J, Lenardo MJ (2006). The Vif and Vpr accessory proteins independently cause HIV-1-induced T cell cytopathicity and cell cycle arrest. Proc Natl Acad Sci U S A.

[B31] Westerhout EM, Vink M, Haasnoot PC, Das AT, Berkhout B (2006). A conditionally replicating HIV-based vector that stably expresses an antiviral shRNA against HIV-1 replication. Mol Ther.

[B32] Berkhout B, Jeang KT (1989). Trans activation of human immunodeficiency virus type 1 is sequence specific for both the single-stranded bulge and loop of the trans-acting-responsive hairpin: a quantitative analysis. J Virol.

[B33] Berkhout B, Klaver B (1993). In vivo selection of randomly mutated retroviral genomes. Nucleic Acids Res.

[B34] Feng S, Holland EC (1988). HIV-1 tat trans-activation requires the loop sequence within tar. Nature.

[B35] Klaver B, Berkhout B (1994). Evolution of a disrupted TAR RNA hairpin structure in the HIV-1 virus. EMBO J.

[B36] Chattopadhyay SK, Morse HCIII, Makino M, Ruscetti SK, Hartley JW (1989). Defective virus is associated with induction of murine retrovirus-induced immunodeficiency syndrome. Proc Natl Acad Sci USA.

[B37] Desfosses Y, Solis M, Sun Q, Grandvaux N, Van Lint C, Burny A, Gatignol A, Wainberg MA, Lin R, Hiscott J (2005). Regulation of human immunodeficiency virus type 1 gene expression by clade-specific tat proteins. J Virol.

[B38] Das AT, Zhou X, Vink M, Klaver B, Verhoef K, Marzio G, Berkhout B (2004). Viral evolution as a tool to improve the tetracycline-regulated gene expression system. J Biol Chem.

[B39] Gibbs JS, Regier DA, Desrosiers RC (1994). Construction and in vitro properties of HIV-1 mutants with deletions in "nonessential" genes. AIDS Res Hum Retroviruses.

[B40] Auersperg N (1964). Long-term cultivation of hypodiploid human tumor cells. J Nat Cancer Inst.

[B41] Das AT, Klaver B, Berkhout B (1999). A hairpin structure in the R region of the Human Immunodeficiency Virus type 1 RNA genome is instrumental in polyadenylation site selection. J Virol.

[B42] Smith SD, Shatsky M, Cohen PS, Warnke R, Link MP, Glader BE (1984). Monoclonal antibody and enzymatic profiles of human malignant T- lymphoid cells and derived cell lines.. Cancer Res.

[B43] Melkonyan H, Sorg C, Klempt M (1996). Electroporation efficiency in mammalian cells is increased by dimethyl sulfoxide (DMSO).. Nucleic Acids Res.

[B44] Jeeninga RE, Keulen W, Boucher C, Sanders RW, Berkhout B (2001). Evolution of AZT resistance in HIV-1: the 41-70 intermediate that is not observed in vivo has a replication defect. Virol.

